# Nanocrystalline Alumina-Zirconia-Based Eutectic Ceramics Fabricated with High-Energy Beams: Principle, Solidification Techniques, Microstructure and Mechanical Properties

**DOI:** 10.3390/ma16082985

**Published:** 2023-04-09

**Authors:** Zhi-Gang Wang, Yun-Zhuo Zhang, Jia-Hu Ouyang, Xi-Wen Song, Min Xie, Ya-Ming Wang, Yu-Jin Wang

**Affiliations:** 1 School of Materials and Metallurgy, Inner Mongolia University of Science & Technology, Baotou 014010, China; 2 School of Materials Science and Engineering, Harbin Institute of Technology, Harbin 150001, China

**Keywords:** alumina-zirconia ceramics, nanoeutectic, eutectic solidification, microstructure

## Abstract

Nanocrystalline alumina-zirconia-based eutectic ceramics fabricated with high-energy beams and composed of ultrafine, three-dimensionally entangled, single-crystal domains are a special category of eutectic oxides that exhibit exceptionally high-temperature mechanical properties, such as strength and toughness as well as creep resistance. This paper aims to provide a comprehensive review on the basic principles, advanced solidification processes, microstructure and mechanical properties of alumina-zirconia-based eutectic ceramics, with particular attention to the status of the art on a nanocrystalline scale. Some basic principles of coupled eutectic growth are first introduced based on previously reported models, followed by concise introduction of solidification techniques and the control strategy of solidification behavior from the processing variables. Then, the microstructural formation of nanoeutectic structure is elucidated with regard to different hierarchical scales, and mechanical properties such as hardness, flexural and tensile strength, fracture toughness and wear resistance are discussed in detail for a comparative study. Nanocrystalline alumina-zirconia-based eutectic ceramics with unique microstructural and compositional characteristics have been produced with high-energy beam-based processes, and in many cases, promising improvements in mechanical performance have been reported as contrasting with conventional eutectic ceramics.

## 1. Introduction

Eutectic materials are a paradigm of composite materials with self-assembled microstructures, on the micro- or nanoscale, whose characteristics are mediated by processing conditions, and these oriented eutectic microstructures are produced in systems that have revealed potential industrial applications in certain key areas of materials technology. Considerable attention was generally concentrated in the past, to a great extent, on metallic eutectic alloys, and the most significant advances associated with the understanding of eutectic growth, microstructure and properties were achieved in these materials. In the case of eutectic ceramics, a relatively systematic investigation was originally published in the early 1960s, and considerable research has been dedicated to the microstructures and properties of eutectic ceramics. Among a large number of eutectic ceramics with attractive properties, a particular interest has been shown in oxide-oxide systems in which alumina-based eutectic in situ composites have attracted extensive attention for potential applications in ultrahigh-temperature structural-functional materials or advanced protective coatings due to their superior specific strength, stiffness and intrinsic thermal stability up to 1773 K near their melting points under an oxidizing atmosphere [1,2,3,4,5,6,7,8].

Alumina-based eutectic ceramics characterized by the coupled growth of two (or more) phases from liquid are particularly intriguing, as they consist of three-dimensionally continuous and entangled monocrystalline Al_2_O_3_ and other oxide monocrystals—ZrO_2_(RE_2_O_3_), RE_3_Al_5_O_12_, REAlO_3_, etc.—where RE = rare earth, yttrium or their combinations [9,10]. These in situ composites have been extensively investigated in the last few decades due to their superior mechanical properties inherent to homogeneous microstructures, their reduced interphase spacing and the presence of large amounts of clean, strongly cohesive interfaces, heretofore unachieved in sintered polycrystalline ceramic materials [11,12]. Furthermore, the intriguing microstructures of regular eutectic ceramics, consisting of ordered 3D arrays of alternating lamellae or fibers, are also prospective materials for a variety of structural and functional applications, such as optical, electronic, magnetic, piezoelectric and ferromagnetic. From the point of view of processing, in situ oxide eutectic composites are often produced, as in metallic systems, with directional solidification of eutectic melts: for example, the Bridgman method; the Czochralski method; the edge-defined, film-fed growth method; and the micro-pulling-down method. It should be noted that the aforementioned processing is extremely restricted to a relatively low temperature gradient and a slow growth rate and usually generates an undesirable microstructure with large interphase spacings in the micron range, which clearly deteriorates properties.

As a result of advances in preparation and characterization, nanocrystalline alumina-based eutectic ceramics with novel and ultrafine microstructures have emerged as an important area of research due to their capability to demonstrate improved and unique properties. Generally, “smaller is stronger” is a paradigm in materials development [13,14]. In this regard, nanoeutectic ceramics exhibit unique combinations of processing characteristics and mechanical and physical properties compared to their coarse-microstructured counterparts, in turn generally resulting from a higher growth rate. In the realm of mechanical properties, especially for alumina-zirconia-based binary or ternary eutectic systems, distinct improvements in both bending strength (up to 4.6 GPa) and moderated toughness (4 MPa·m^1/2^) have been achieved for rods of Al_2_O_3_-Y_3_Al_5_O_12_-Zr_2_O_3_(Y_2_O_3_) grown at a superior rate of 1200 mm·h^−1^ with a laser-heated floating-zone method [13]; this is more than twice as high as the maximum value reported in directionally solidified eutectic (DSE) oxides of the Al_2_O_3_-Y_3_Al_5_O_12_-ZrO_2_(Y_2_O_3_) family [1,15,16]. Furthermore, the Al_2_O_3_-ZrO_2_(Y_2_O_3_) eutectic system is of particular interest due to its high strength over a wide temperature range, its toughness, its creep resistance and its chemical stability. From a theoretical point of view, solidification morphology depends upon chemical composition, temperature gradient and growth rate, hence bulk nanostructured eutectic ceramics can be generally produced with rapid solidification from melting. However, it is usually difficult for oxide ceramics to achieve both a high growth rate and a large temperature gradient via the conventional solidification process due to their intrinsic high melting points, phase stability and lower thermal and electrical conductivity. Consequently, minor efforts have been devoted to the area of alumina-zirconia-based nanoeutectic ceramics as compared to microscale DSE oxides. Therefore, this is a relatively young research field, and there continually are great gaps in its comprehension.

The present contribution aims to review some of these developments and is organized as follows: Firstly, background on alumina-zirconia-based nanoeutectic ceramics is presented, and then Section 2 is dedicated to the theoretical basis of coupled eutectic growth, which is necessary to obtain insight into the relationships between processing conditions and microstructure parameters. Section 3 provides a comparative analysis of different rapid-solidification processes of alumina-zirconia-based nanoeutectic ceramics, along with a clear understanding of advantages and disadvantages. Section 4 presents a variety of eutectic microstructures existing in these ceramics and clarifies in detail the mechanisms of microstructure formation, with particular attention to the microstructural features of eutectic ceramics existing on three different size scales. The basic mechanical properties of these ceramics (e.g., hardness, flexural and tensile strength, toughness, wear resistance, etc.) and their relationship to the nanostructuring of eutectic microstructures are analyzed in the last section.

## 2. Theoretical Growth Models of Nanocrystalline Alumina-Zirconia Eutectic Ceramics

It is worth to noting that two classical models have been proposed, in the literature, on the theoretical aspects of eutectic solidification: the Jackson and Hunt (JH) model [17] established the classical theory of regular eutectic growth under restrictions of low velocities only, and the Trivedi, Magnin and Kurz (TMK) model [18] extended the JH model to treat regular eutectics with high growth velocities via relaxing some of the major simplifying assumptions made by the JH model. The objective of this section is to introduce necessary theoretical background for the subsequent discussion of nanoeutectic ceramic microstructures and their properties. Hereby, special focus is put on the basic principles applicable to the growth of alumina-based nanoeutectic ceramics.

### 2.1. Growth Undercooling

The eutectic solidification process is governed by extraction of heat from the melt system, and if the heat extraction rate is externally determined, then the growth rate of a eutectic is fixed and the interlamellar/rod spacing is constant. Therefore, there must exist an undercooling below the equilibrium eutectic temperature to drive a solid/liquid (S/L) interface at the specified growth rate. In general, interface undercooling is controlled essentially by the interplay of solute (or heat) diffusion processes and capillarity forces to determine the spacing of precipitating phases. Particularly, the difference between the local actual temperature at the S/L interface (*T_l_*) and the eutectic temperature (*T_e_*) is made up of three contributions:

(1) $$\Delta T=T_{e}-T_{l}=\Delta T_{c}+\Delta T_{r}+\Delta T_{k}$$

where Δ*T_c_* is chemical (or solute) undercooling due to a locally slight departure of the equilibrium composition from the eutectic composition (*C_e_*), leading to a proportional departure of the equilibrium temperature from the eutectic temperature. In this regard, this term has only been taken into consideration in solid solutions with distribution coefficients of less than unity, which can be written as:

(2) $$\Delta T_{c}=m_{i}\left[C_{e}-C\left(x\right)\right]\;i=\alpha ,\;\beta$$

where *m_i_* is the liquidus slope given by the phase diagram and *C*(*x*) is the composition of the S/L interface at *x*. The curvature undercooling, Δ*T_r_*, represents the Gibbs-Thomson capillary effect of S/L-interface curvature on the equilibrium temperature between the solid and liquid phases and can be expressed as

(3) $$\Delta T_{r}=\kappa \Gamma_{i}\;i=\alpha ,\;\beta$$

where Γ and *κ* are the Gibbs-Thomson coefficient and the local interface curvature, respectively. The third contribution, Δ*T_k_*, is the kinetic undercooling required by the kinetic process of atom attachment to the interface, which stands for the difference between the chemical potentials of the solid and liquid phases at the interface. In the case of nanoeutectic ceramics, it is worthy of mention here that a large kinetic term is significant when eutectic ceramics grow under rapid solidification conditions or contain phases with high entropy of melting.

### 2.2. Interface Thermodynamic Equilibrium

By following the procedures given by JH and TMK, the relationship between the growth rate, *V*; the lamellar/rod spacing, *λ*; and the average undercooling, Δ*T*; is given as [17,18]

(4) $$\Delta T=K_{1}V\lambda +K_{2}/\lambda$$

where *K_1_* and *K_2_* are no longer regarded as constants under the condition of a high growth rate [19];

(5) $$K_{1}=\frac{m_{v}\Delta C_{0}^{{}_{v}}}{D}\frac{P}{f_{\alpha }f_{\beta }}$$

(6) $$K_{2}=2m_{v}\sum_{i}\left(\frac{\Gamma_{i}\sin\theta_{i}}{m_{i}^{v}f_{i}}\right)\;i=\alpha ,\;\beta$$

(7) $$P=\sum_{n=1}^{\infty }{\left(\frac{1}{n\pi }\right)}^{3}{\left[\sin(n\pi f)\right]}^{2}\frac{p_{n}}{\sqrt{1+p_{n}^{2}}-1+2k_{v}}$$

where the angles, *θ_i_*, are the contact angles between the two solid phases and the liquid at the three-phase junction; $m_{i}^{v}$ is the effective liquidus slopes for the two phases; the parameter *k_v_* is the distribution coefficient under high growth rates, the detailed description of which can be found in the literature [19]; *p_n_* = 2*n*π/*p*, where *p* is the eutectic Péclet number equal to *Vλ*/2*D*; *m_v_* is the effective mean slope of the liquidus lines, as a function of the growth rate; *D* is the interdiffusion coefficient in the liquid; *f_α_* and *f_β_* are the volume fractions of the *α* and *β* phases, respectively; and $\Delta C_{0}^{{}_{v}}$ is the compositional range between *α* and *β* at the growth temperature in simple eutectic ceramics between two constituents, A and B. Here, rapid solidification effects enter through the terms of $m_{i}^{v}$ and *k_v_*. For regular lamellar/fiber eutectic ceramics, this has been demonstrated to grow very close to extremum via stability analysis of the eutectic interface [18]. Thus, through adopting extremum criteria (e.g., minimum undercooling), which is a suitable portrayal of experimentally observed average growth behavior, and through setting ${\left(\partial \Delta T/\partial \lambda \right)}_{V}=0$, the following relationships are obtained:

(8) $$\lambda^{2}V=K_{2}/K_{1}$$

(9) $$\Delta T/\sqrt{V}=2\sqrt{K_{1}K_{2}}$$

(10) $$\lambda \Delta T=2K_{2}$$

In the case of irregular eutectic ceramics, growth behavior is generally far from an extremum condition. However, at this point, it can be assumed that growth occurs at a spacing of $\lambda =\phi \lambda_{ex}$, where *λ_ex_* is the spacing corresponding to the minimum undercooling for a given growth rate and ϕ is a constant reflecting the spacing adjustment mechanism [20]. In this regard, one can describe the relationship using Equations (8) through (10), and therefore the following relationships are obtained:

(11) $$\lambda^{2}V=\phi^{2}K_{2}/K_{1}$$

(12) $$\Delta T/\sqrt{V}=\left(\phi +1/\phi \right)\sqrt{K_{1}K_{2}}$$

(13) $$\lambda \Delta T=\left(\phi^{2}+1\right)K_{2}$$

**Figure 1:**
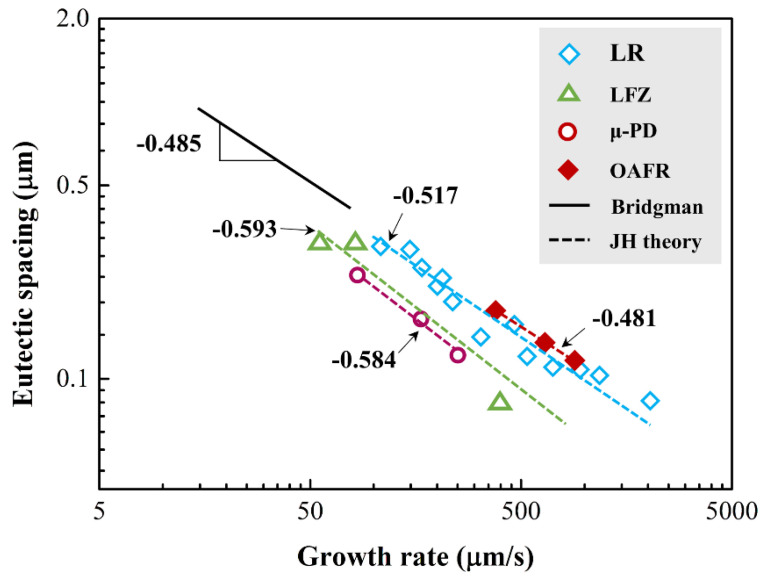
Variations of eutectic spacings with the corresponding growth rates on a logarithmic scale for Al_2_O_3_-ZrO_2_(Y_2_O_3_) nanoeutectic ceramics induced via various solidification processes. Reproduced with permission from Reference [33], Copyright © 2018 Elsevier Ltd.

Based on this, it has been well-established that variation of lamellar/fiber spacing versus growth rate is essentially linear on the logarithmic scale, as described by Equations (8) and (11) in the previous Section 2. As can be seen from Figure 1, the data from the Al_2_O_3_-ZrO_2_ eutectic system fit straight lines, and linear regression analysis gave the proportionality equation as $\lambda =kV^{-m}$ (for a constant, *G*). These were reported for regular microstructures as well as for complex regular ones, the latter measured in the ordered areas inside the colonies. The dependence of the *λ* values of Al_2_O_3_-ZrO_2_ eutectic ceramics on the growth rates is exponential, and the value of exponent *m* relating to the growth rate for *λ* were found to be close to the value of 0.50, predicted by the JH eutectic theory. In general, the experimental measurements of regular eutectic spacing of alumina-based nanoeutectic ceramics obey a relationship where *λ*^2^*V =* constant, with a substantial variation in the proportionality constant of 1.0 μm^3^·s^−1^ for Al_2_O_3_-ZrO_2_ [21,22,23,24]. Note, however, that some rapidly solidified eutectic systems (Al_2_O_3_-GdAlO_3_ [8,25,26], Al_2_O_3_-GdAlO_3_-ZrO_2_ [27,28], Al_2_O_3_-Er_3_Al_5_O_12_ [29,30], Al_2_O_3_-Y_3_Al_5_O_12_ [31,32], etc.) appear to deviate from this behavior due to high Péclet numbers.

## 3. Solidification Techniques for Nanocrystalline Alumina-Zirconia Eutectic Ceramics

**Figure 2:**
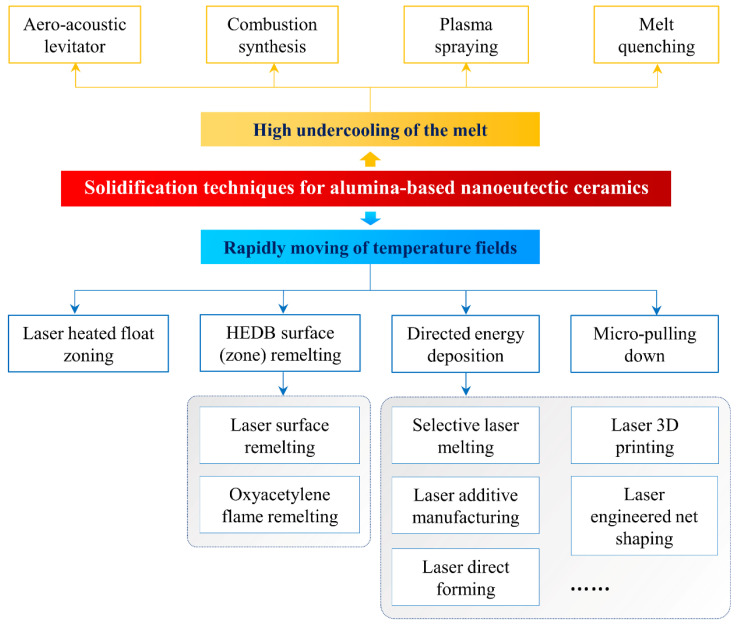
Classification of processing methods of alumina-based nanoeutectic ceramics.

There are several available methods, which have been developed in the past, that can be adopted to solidify alumina-based nanoeutectic ceramics in the laboratory, such as laser-heated float zoning (LFZ) [8,13,14,26,28,29,30,32,34,35,36,37,38,39,40,41,42,43,44,45,46,47,48,49,50,51,52,53], micro-pulling-down (μ-PD) [23,54], aero-acoustic levitators [55], high-energy density beam (HEDB) (laser or oxy-acetylene flame) surface (zone) (re)melting [24,33,56,57,58,59,60,61,62,63,64,65,66,67,68], plasma spraying [69,70], directed energy deposition (DED) [21,22,25,27,31,71,72,73,74,75,76,77,78,79,80,81,82,83], melt quenching [32,52,84,85], combustion synthesis [70,86,87], etc. Figure 2 illustrates different methods applied for preparation of alumina-based nanoeutectic ceramics. Generally, all these methods can be categorized into two main types, which exhibit distinct differences in processing parameters: (i) rapidly moving temperature fields through means of a high-energy heat source such as an oxy-acetylene flame, a laser, or an electron beam, and (ii) high undercooling of the melt, which can be provided by a large cooling rate or can be produced by the absence of efficient heterogeneous nucleates. The speed of the S/L interface advancing, *V*, is the most important variable in affecting the solidification behavior. The temperature gradient, *G*, in the liquid ahead of the advancing S/L interface plays a less important role in determining microstructure evolution processes. The further important variables of both the imposed cooling rate, d*T*/d*t* (in directional growth, is equivalent to *GV*), and the *G*/*V* ratio control the stability of a planar interface at low rates (constitutional undercooling). The following illustrates some techniques, based on a high-energy-beam remelting process, that have been most extensively used for fabrication of the alumina-zirconia-based nanoeutectic ceramics.

### 3.1. Laser-Heated Float-Zoning Technique

**Figure 3:**
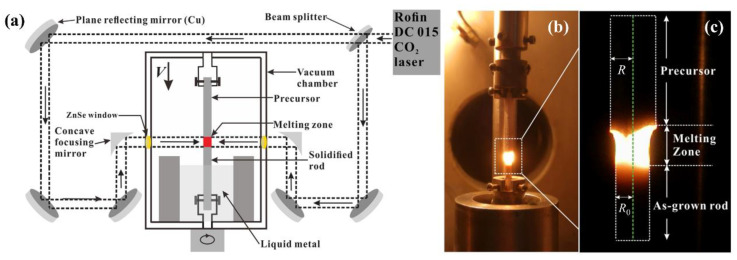
Diagrammatic view of the laser floating-zone melting apparatus (**a**); photograph of the procedure of an Al_2_O_3_-based, directionally solidified eutectic ceramic specimen (**b**); and principle of the capillary stable-crystal-growth condition (**c**). Reproduced with permission from Reference [53], Copyright © 2019 Elsevier Inc., and Reference [90], Copyright © 2021, Elsevier.

This technique is based on continuous transport of the melt through micro-zone melting in a capillary tube maintained by surface tension between the crystal and the precursor, with steeper thermal gradients and consequently higher growth rates; Figure 3 is a diagrammatic view of the heater-arrangement laser floating-zone system. The fundamentals of fiber crystal growth from free-melt meniscus, i.e., the melt zone, are well described in a review paper delivered by Rudolph et al. [88]. Afterward, the LFZ method, which was originally developed by Sayir et al. [34] and Peña et al. [36], is used to fabricate nanostructured Al_2_O_3_-ZrO_2_(Y_2_O_3_) eutectic bars with a 2 mm diameter each at a variable growth rate in the range of 10–1500 mm·h^−1^. In this case, the maximum thermal gradient at the interface was determined to be about 8 × 10^5^ K·m^−1^. The axial thermal gradient for the small rod diameters at the interface of *z* = 0 can be estimated with, for example, the following expression proposed by Brice [89]:

(14) $${\left(dT/dz\right)}_{z=0}\approx \sqrt{\frac{2h}{R}}\left(T_{m}-T_{0}\right)$$

which is essentially independent of *r* and decreases with increasing rod diameter. Similarly, the radial thermal gradient is independent of the distance to the rotation axis, and

(15) $${\left(dT/dr\right)}_{z=0}\approx r\sqrt{\frac{h}{2R}}\left(dT/dz\right)$$

where *h* is the cooling constant; *T_m_* and *T*_0_ are the melting temperature and the ambient temperature, respectively; and *R* is the rod diameter. Nevertheless, geometry of samples remains one of the primary obstacles toward practical application of this technique, and cylinders are restricted to maximum diameters that depend on the material properties, as do the growth rate and thermal gradients at the interface of solidification. Establishment of a stable capillary growth condition is crucially important in controlling the dimensional stability of a growing fiber from a shaped meniscus. Based on the mass conservation law, the condition for steady-state growth of a fiber material with a constant diameter was established [88]:

(16) $$R=R_{0}\sqrt{\frac{\upsilon_{0}}{\upsilon }}$$

where *R* and *R*_0_ are the radii of a grown fiber and a shaper (i.e., capillary or feed rod), respectively, and υ and $\upsilon_{0}$ are the velocities of the grown fiber and the feeding rod or shaper, respectively. Dense and defect-free bar-shaped samples can be achieved via applying the LFZ method, although they in general can be used for mechanical testing or physical characterization (i.e., mechanical, ionic or electronic conduction; light-guiding effects; etc.), presenting a limitation with regard to some potential applications requiring large-scale eutectic structured surfaces.

### 3.2. High-Energy-Beam Surface (Zone) (re)Melting Technique

**Figure 4:**
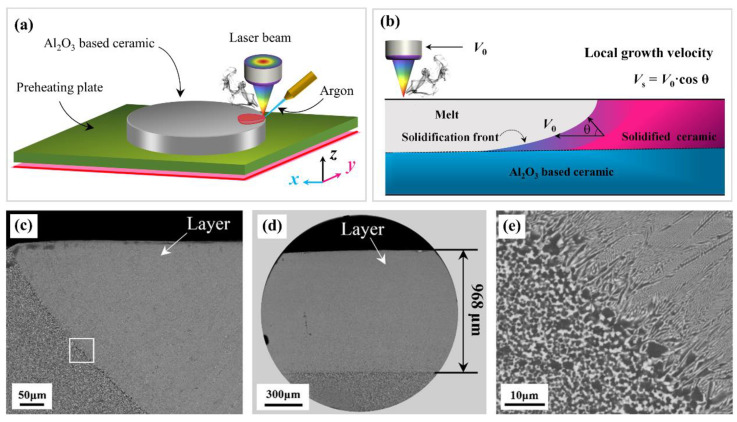
Schematic illustrations of (**a**) the setup for laser remelting and (**b**) the solidification process for surface nanostructuring of an Al_2_O_3_-ZrO_2_ ceramic with a eutectic composition. The local growth rate (*V*_s_) is related to the laser scanning rate (*V*_0_) with the expression of $V_{s}=V_{0}\cdot \cos\theta$, where *θ* is the tangent angle of the solidification front. Transverse cross-sections of nanostructured Al_2_O_3_-ZrO_2_ eutectic layers via laser remelting at a heat input of 416.7 kJm^−1^ (**c**,**d**) are different regions of a single laser-melted track. (**e**) The enlarged view of the square region marked in (**c**). Reproduced with permission from Reference [67], Copyright © 2019 Elsevier B.V.

**Figure 5:**
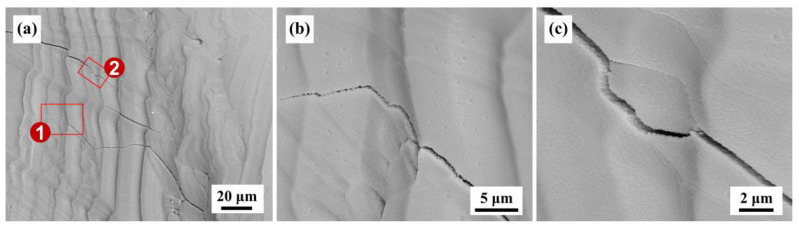
Thermal-stress-induced crack deflection at raised striations of wrinkle-textured, nanostructured Al_2_O_3_-ZrO_2_ eutectic ceramics formed by the oxyacetylene flame remelting (OAFR) process; ①② are the visible cracks and the regions of ① and ② marked by red rectangles in (**a**) are correspondingly shown in (**b**,**c**) [65], Copyright © 2018 The American Ceramic Society.

High-energy-beam surface treatment, more specifically high-energy-beam (re)melting, is techniques toward improving the wear, fatigue, corrosion, impact resistance and dimensional stability of mechanical parts. Of the various high-energy-beam sources that have been adopted in manufacturing of alumina-zirconia-based eutectic ceramics are lasers, electron beams and oxyacetylene flames. In these processes, as a moving, high-energy beam irradiates on the ceramic plate, absorbed energy is conducted from the surface into the bulk of the ceramic, and then the surface region under the high-energy beam is melted to form the deep melt pool, followed by rapid solidification, as depicted in our previous work (Figure 4a in Ref. [67] in the case of laser remelting). The trailing edge of the moving melt pool is the solidification front where microstructural evolution occurs. From a theoretical point of view, the melting-solidification behavior within the melt pool is mainly governed by high-energy processing parameters such as beam power, scanning speed and interaction time, and hence on ceramic properties such as optical properties, thermal diffusivity and thermal expansion. From a process point of view, scanning speed is the most dominant parameter, followed by high-energy-beam power such as laser irradiation, both of which are highly controllable in determining the melting and cooling rates of the molten pool and therefore the crystal growth rate. As a rule of thumb, the local growth rate (*V*_s_) of a crystal is equal to the product of the laser scanning speed (*V*_0_) and the cosine of the tangent angle, *θ*, as depicted in Figure 4b. The transverse cross-sections of nanostructured Al_2_O_3_-ZrO_2_ eutectic layers processed with laser remelting are shown in Figure 4c–e. The shape and dimensions of the melt pool depend markedly upon the process parameters, and the full details are available in our previous publications [67,68]. To avoid cracking induced by thermal shock, one of the obvious strategies is to perform preheating of the substrate to be treated [60]. Finally, it is worth noting that the distinction between the heating sources employed lies mainly in the aspects of physical and/or chemical interaction behavior, such as heat absorption, heat radiation, ablation damage, shockwave effects, etc. In particular, shockwave effects can be employed to produce wrinkle-textured surfaces, which play a dominant role in affecting crack deflection, as is evidenced in Figure 5. From a nanomanufacturing point of view, the advantages of laser and electron-beam irradiation lie in the inherent efficiency of beam generation and the relative compactness of the beam source compared with an oxyacetylene flame source. This allows laser- and electron-beam processing techniques to be potentially scaled to an advanced ceramic industrial process.

### 3.3. Laser Additive Manufacturing

Laser-based additive manufacturing (AM) processes are characterized by layer-by-layer construction or buildup of parts via laser fused deposition modeling without the use of part-specific tooling. Compared with traditional manufacturing methods, laser-based AM processes have several advantages. In general, the laser beam concentrates the heat to a relatively small localized area of the part based on the laser spot size. Hence, high precision can be achieved, with excellent metallurgical bonding between layers and relatively low distortion because of the low heat input of the laser. In addition, due to the high-energy density of laser beams, a high-temperature gradient can be achieved in the melt pool, and thus, it is feasible to be applied to obtain a finer solidification microstructure and to process difficult-to-machine refractory materials. Furthermore, laser AM provides significant opportunities and economic benefits for design of complex geometries and even for one-step manufacturing of composite materials or functional gradient-material components.

To date, a variety of laser additive manufacturing techniques, including laser-directed energy deposition (LDED) [82,83,91], selective laser melting (SLM) [25,27,92], the laser-engineered net-shaping technique (LENS) [77,78] and laser 3D printing [31,80,93], have been presented as a new paradigm for manufacturing of alumina-zirconia-based nanoeutectic ceramics. However, the most serious drawbacks of laser-based AM in general are associated with the presence of unacceptable residual stress and defects, such as residual pores, distortion and even cracking and delamination in the case of inherently brittle ceramics, which impose certain restrictions on the size and geometry of bulk nanoeutectic ceramics. In this regard, the current studies reported have been mainly restricted to thin-walled or long, cylinder-shaped, alumina-zirconia-based eutectic ceramics: for example, Al_2_O_3_-GdAlO_3_-ZrO_2_ [27,82,83,92,93], Al_2_O_3_-ZrO_2_ [77,80,91], Al_2_O_3_-GdAlO_3_ [25], Al_2_O_3_-YAG-ZrO_2_ [78], Al_2_O_3_-YAG [31], etc. Detailed descriptions of the microstructures and crystallography of alumina-zirconia-based nanoeutectic ceramics fabricated with laser AM can be found in the recently published literature [94].

### 3.4. Summary of Solidification Techniques

Among the former methods, laser-heated float zoning, high-energy-beam surface melting and directed energy deposition are the current prevailing approaches for producing alumina-based nanoeutectic ceramics. It is worth noting that the common characteristic of these three methods is the fact that rapid melting-solidification is achieved based on thermal effects caused by high-energy density beams. More specifically, the main advantages of these methods are (1) minimizing possible sources of contamination with foreign impurities and allowing for very high melt temperatures in the absence of any container or die, and (2) feasibility to produce a planar and stable solidification front due to an extremely fast cooling rate and a steep temperature gradient at the S/L interphase, hence preventing constitutional undercooling and cellular growth and allowing for finer and homogeneous eutectic growth rates. In summary, the high-energy-beam surface (zone) (re)melting technique is a suitable approach for rapid manufacturing of nanostructured eutectic surface layers of ceramics without affecting bulk ceramic behavior. The laser-heated float-zoning technique can produce cylindrical alumina-zirconia-based nanoeutectic ceramic fibers with a maximum diameter in the order of several millimeters, which is not suitable for many applications, however, and severe restrictions are found when large-scale surfaces of eutectic ceramic components are required. Relatively speaking, laser-based additive manufacturing processes are not subject to the constraints associated with the aforementioned methods, and they provide significant opportunities for design of novel geometries and complex internal structures of large-size bulk nanoeutectic ceramics.

## 4. Solidification Microstructures of Nanocrystalline Alumina-Zirconia-Based Eutectic Ceramics

Due to the fact that eutectic solidification is characterized as simultaneous crystallization of more than one phase from a liquid, eutectic microstructures can exhibit a wide variety of complex geometrical arrangements of eutectic phases, which offers great potential for tailoring structures and properties, especially for oxide eutectic ceramics. Generally, the microstructural features of eutectic ceramics exist on three different size scales that can be readily distinguished in alumina-based nanoeutectic ceramics based on the nucleation-growth process of eutectic phase microstructure, grain structure and colony structure, as do directionally solidified eutectic ceramics [95]. Other important aspects are size, shape and crystallographic orientation, as well as the morphologies and nature of the interfaces between eutectic phases, colonies and grains. The microstructures and crystallography of directionally solidified oxide eutectic ceramics were reported by LLorca et al. [96], Merino et al. [97] and Lakiza [98]. Those previous results were taken into consideration here, but this discussion is mainly focused on alumina-zirconia-based nanoeutectic ceramics with regard to the abovementioned aspects.

### 4.1. Eutectic Phase Microstructure

**Figure 6:**
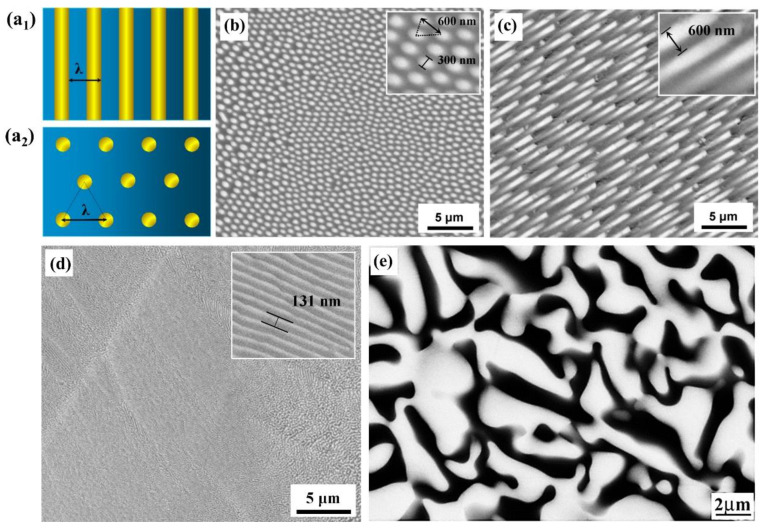
Schematic geometries of ideal cross-sections and eutectic spacing of the (**a_1_**) lamellar and (**a_2_**) fibrous eutectic structures; back-scattering electron images showing the microstructure of the Al_2_O_3_-ZrO_2_ nanoeutectic of (**b**) the regular fibrous eutectic structure, (**c**) the longitudinal section of (**b**) and (**d**) the regular lamellar eutectic structure; and (**e**) the back-scattering electron image showing the microstructure of the Al_2_O_3_-GdAlO_3_ irregular eutectic. Reproduced with permission from Reference [100], Copyright © 2018 Elsevier Ltd., Reference [33], Copyright © 2018 Elsevier Ltd. and Reference [46], Copyright © 2018 Elsevier Ltd. and Techna Group S.r.l.

Within one eutectic grain, the microstructure consists of three-dimensional and continuous interconnected networks of monocrystalline eutectic phases, which can be considered the smallest component units in solidified eutectic ceramics. In general, alumina-based nanoeutectic ceramics can be distinguished as two types of eutectic microstructures: normal and anomalous. The normal eutectic structure is either lamellar, where in both plate-like phases, the growth direction is contained in the interfacial boundary, or fibrous, in which one phase is rod-like and embedded in a single alumina matrix of the second phase, either perovskite or garnet, with the fiber axis parallel to the growth direction [95]. In cases of ternary systems, cubic zirconia is added as a third phase. In common with normal eutectic materials, the microstructures of eutectic phases are influenced by volume fraction and growth velocity as well as the anisotropic properties of the eutectic phases (such as growth rate, surface energy and thermal conductivity) and small quantities of impurities. As a rule of thumb [99], one can suppose that when the minor phase is present in a small volume fraction (*f* < 0.28), the eutectic will probably be fibrous (e.g., Al_2_O_3_-ZrO_2_ [51,74,86,87,100], Al_2_O_3_-GdAlO_3_ [8,26,42]) (Figure 6a1–c). If it is between 0.28 and 0.5, there is a tendency to prefer the lamellar structure (Figure 6a2) (e.g., Al_2_O_3_-ZrO_2_ [73,75], Al_2_O_3_-Y_3_Al_5_O_12_ [32]) (Figure 6d).

Most regular eutectic oxide ceramics follow the phase-volume rule. Nevertheless, regular fibrous and lamellar microstructures are the exception rather than the rule in alumina-based nanoeutectic ceramics due to rapid solidification growth and the intense tendency of most oxide crystals to grow along certain crystallographic planes, for example, the faceting of Al_2_O_3_ presenting a triangular [14,28,48,78,101] or complex geometrical network [47,48]. When faceting occurs, the eutectic morphology often tends to be irregular, and this is particularly true in the cases of alumina-based nanoeutectic ceramics—Al_2_O_3_-GdAlO_3_ [8,26], Al_2_O_3_-GdAlO_3_-ZrO_2_ [27,28,81,82,83], Al_2_O_3_-Er_3_Al_5_O_12_ [29,53,62], Al_2_O_3_-Er_3_Al_5_O_12_-ZrO_2_ [47,50], Al_2_O_3_-Y_3_Al_5_O_12_ [63] and Al_2_O_3_-Y_3_Al_5_O_12_-ZrO_2_ [41,46,78]—as shown in Figure 6e. The anisotropy degree of the growth behavior of eutectic phases can be predicted with Jackson’s roughness parameter, defined as $\alpha =\xi \Delta S_{m}/R$, where Δ*S*_m_ is melting entropy, *R* the gas constant and *ξ* a crystallographic parameter close to but less than unity. Values of *α* that are less than 2 generally imply a tendency toward nonfaceted growth, while higher *α*-values indicate that faceted growth will occur with the rate of growth, limited by the rate of nucleation. Especially for oxide ceramics with high melting-entropy values, the presence of faceted crystalline phases is a rule rather than an exception in DSE oxide systems [101]. For example, a high-melting-entropy phase may become nonfaceted when it grows under a sufficiently high driving force, such as high undercooling.

**Figure 7:**
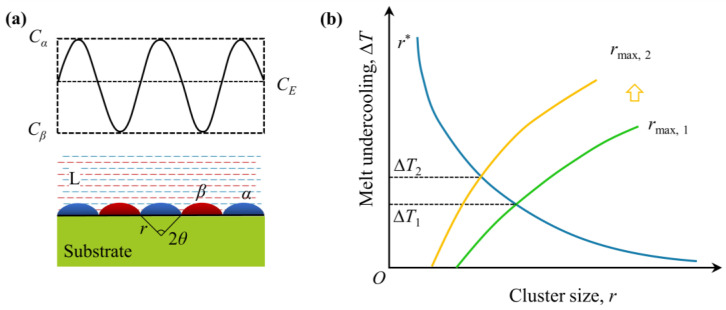
Constituent fluctuation (**a**) and relationships among heterogeneous nucleation undercooling, Δ*T*; critical nucleation size, (r*); and (**b**) maximum crystal embryos (*r*_max_), where the upward arrow indicates an increase in critical size [102]. Copyright © 2022 Elsevier Ltd.

Another noteworthy aspect of eutectic-phase microstructure to be discussed is interlamellar or interfiber spacing, which can drastically affect the properties of a ceramic. Based on Equation (8) through (10), enhanced undercooling and a rapid growth rate are the essential mechanisms associated with microstructural refinement, which can be well-revealed with classical heterogeneous nucleation theory [33]. It is well-established that there generally exist short-range ordered clusters with different sizes in the melt due to constituent fluctuation, as shown in Figure 7a. However, the formation of a critical crystal embryo of size r* needs to overcome its critical activation energy (ΔG*), as described in

(17) $$\Delta G^{*}=\frac{16\pi \gamma^{3}}{3\Delta G_{v}{}^{2}}\left[\frac{(2+\cos\theta ){\left(1-\cos\theta \right)}^{2}}{4}\right]$$

(18) $$r^{*}=2\gamma /\Delta G_{v}=\frac{2\sigma T_{e}}{L_{m}\Delta T}$$

where *γ* is the interfacial energy of radius r*, *θ* is the wetting angle and Δ*G*_v_ is the Gibbs free energy difference between the solid and the liquid phases. To substitute Equation (18) into Equation (17) yields

(19) $$\Delta G^{*}=\frac{4\pi \gamma }{3}{\left(r^{*}\right)}^{2}f(\theta )$$

where *f*(*θ*) = (2 + cos*θ*) × (1 + cos*θ*)^2^/4 as the wetting-angle factor. Obviously, when the radius of the maximum crystal embryos, *r*_max_, decreases from *r*_max_,_1_ to *r*_max_,_2_, as a result, a larger Δ*T* value will be needed in order to meet the nucleation criteria of *r*_max_ ≥ r*, as schematically illustrated in Figure 7b. Consequently, nucleation undercooling (Δ*T*) is required due to enhanced formation of critical crystal embryos. Furthermore, the heterogeneous nucleation rate (*I*) is exponentially related to Δ*T*:

(20) $$I=I_{0}\cdot \exp\left(-\frac{16\pi \sigma^{3}{\left(T_{l}\right)}^{2}}{3{\left(\Delta H\right)}^{2}{\left(\Delta T\right)}^{2}kT}f\left(\theta \right)\right)\cdot \exp\left(-\frac{Q}{RT}\right)$$

where *I*_0_ is a pre-exponential factor, *T_l_* is the liquidus temperature, Δ*H* is the latent heat of fusion, *k* is the Boltzman constant, *R* is the gas constant and *Q* is the activation energy of diffusion. Note that the relationship between undercooling and growth rate (*V*) is given in [28]:

(21) $$\Delta T/\sqrt{V}=2\sqrt{K_{r}\cdot K_{c}}$$

Obviously, increasing Δ*T* has the effects of markedly increasing $I\propto e^{-\Delta T^{-0.5}}$ and the growth rate, $V\propto \Delta T^{2}$, which are the essential mechanism associated with formation of refined eutectic microstructures. In motivation by the above discussion, in the case of high-energy density beam processes, appropriate prolonged irradiation time or slowing down of scanning speed is essential for fabrication of nanostructured eutectic ceramics.

### 4.2. Eutectic Grain Structure

Each eutectic grain originating from one nucleus represents a eutectic nucleation event and then continues to grow in an essentially independent manner, analogous to grains in a polycrystalline ceramic. The size and shape in addition to the morphology of each eutectic grain have great effects on the properties of eutectic ceramics. In order to obtain nanoeutectic microstructures, a high cooling rate must be employed, which implies a far-from-equilibrium eutectic solidification. Consequently, a mixture of both growth forms together, dendritic and eutectic, often exists, although the ceramic is of eutectic composition. Therefore, it is necessary to adjust the starting composition in order to obtain a dendrite-free eutectic microstructure. A eutectic grain originates from a single nucleus, and controlling grain number and size is of utmost importance in tailoring the scale of any eutectic microstructure. The growth behavior of eutectic grains is generally governed by solute segregation and imposed thermal conditions as well as the interface-energy anisotropy of the component phases. In the case of directional solidification, eutectic grains are usually nucleated randomly at the initial stage of the solidification process, are always elongated in the heat-flow direction and usually grow in a direction that is antiparallel to that of the heat flow, as presented in our previous work (Figure 8 in Ref. [65]). Furthermore, growth of columnar eutectic grains exhibits a tilted orientation feature and is deviated from the heat-flow direction to the preferred growth direction, as is the case of high-energy density beam melting, as demonstrated by Wang et al. [64], Su et al. [42], Gurauskis et al. [60] and Liu et al. [27]. In equiaxed solidification, eutectic grains nucleate randomly and then continue to grow in an essential spherical form, as is the case in an undercooled melt, some typical cases of which were presented by Li et al. [55,67,68].

### 4.3. Eutectic Colony Structure

**Figure 9:**
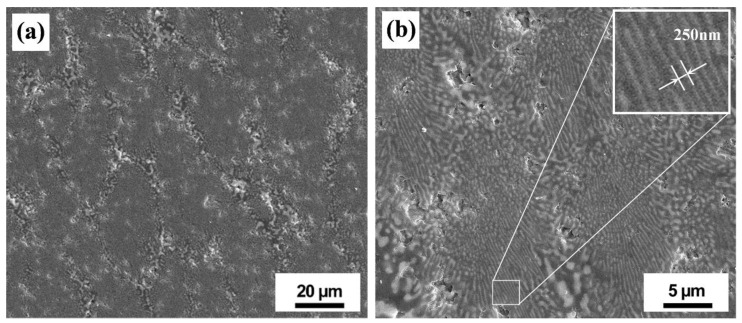
The back-scattering electron image showing the microstructure of the Al_2_O_3_-ZrO_2_(Y_2_O_3_) nanoeutectic: (**a**) typical colony eutectic and (**b**) enlarged view of (**a**). Reproduced with permission from Reference [100], Copyright © 2018 Elsevier Ltd.

**Figure 10:**
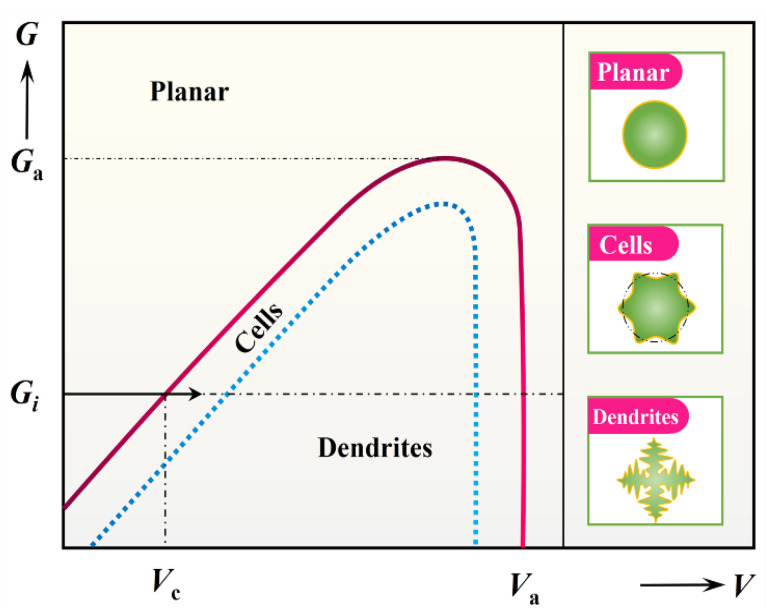
The evolution of S/L interface morphology during equiaxed solidification, where G and V are the critical temperature gradient and the critical growth rate for absolute stability, respectively. Reproduced with permission from Reference [67], Copyright © 2019 Elsevier B.V.

Eutectic colony structure is formed via breakdown of the planar growth front of a lamellar or rod eutectic structure into a cellular or colony structure in which a cell can span several hundred lamellae, as described by Elliott [103]. Eutectic colony structure is more generally presented in the microstructures of alumina-based nanoeutectic ceramics. This is due to constitutional undercooling that develops because of the solute pile-up in front of the S/L interface, formed as a result of solute rejection by both eutectic phases into the liquid when the distribution coefficients are less than unity, as occurs in the Al_2_O_3_-ZrO_2_(Y_2_O_3_) (labeled as Al_2_O_3_-YSZ)-based eutectic system [22,39,51,100], which can be seen in Figure 9 [100], where zirconia is a typical solid-solution phase, as in the case of other rare-earth-doped zirconia. These cells generate this subgrain structure within eutectic grains when the eutectic phases grow perpendicularly to the cellular S/L interface. To put it simply, the colony structure can be rigidly regarded as an aspect of the cell boundary, which can be presented in forms of faceted [37,104,105], spherical [39,51,58], ellipsoidal [27,51,61] or even more complex shapes [25,34,36,82,106]. Eutectic colonies formed as a consequence of the presence of constitutional undercooling at the interface, and their shapes, will be governed by an interplay effect of crystallographic features, thermal diffusion and surface energy considerations. The growth behavior of faceted eutectic cells is analogous to that of classical nonfaceted eutectic phases, but the shapes of the cells will be determined by the orientation of the faceting planes with respect to the growth direction. From a process point of view, a smaller growth rate (*V*) or a larger imposed temperature gradient (*G*), namely a smaller *G/V* value, can prevent or alleviate the occurrence of constitutional supercooling ahead of the S/L interface, consequently preventing growth-interface breakdown and cellular breakdown, as in the case of a rapidly solidified Al_2_O_3_-ZrO_2_(Y_2_O_3_) nanoeutectic, illustrated in Figure 10 [67].

### 4.4. Summary of Solidification Microstructures

With regard to the microstructural features of eutectic alumina-zirconia-based nanoeutectic ceramics, three different size scales can be distinguished: eutectic-phase microstructure, colony structure and grain structure. From a processing perspective, a microstructural tailoring scheme of alumina-zirconia nanoeutectic ceramics can be proposed based on the three different abovementioned size scales. In general, refinement of the constituent phases can be achieved via increasing the cooling rate and/or enhancing nucleation undercooling during solidification. However, the size, morphology and boundary regions of eutectic grains are more challenging to control than those of eutectic phase tailoring, which is governed by the synergistic interplay between the growth rate, the thermal gradient and solute distribution as well as heat transfer. Nevertheless, nanostructuring of eutectic ceramics can indeed decline or eliminate disordered intercellular boundaries, which are regarded as critical solidification defects and are the main obstacles that need to be overcome.

## 5. Mechanical Properties of Nanocrystalline Alumina-Zirconia Eutectic Ceramics

Alumina-based eutectic ceramics are attractive due to their inherent thermochemical stability in high-temperature oxidizing environments, which can retain adequate strength even in oxidizing media above 1650 °C. Moreover, there are other potential functional applications derived from the unique regular and ordered microstructures of eutectic ceramics, such as selective transport of light, heat and electricity, among others. However, two obstacles, fabrication of large-scale components with complex geometries and excellent tradeoff balancing between strength and ductility, limit the engineering applications of alumina-based eutectic ceramics. A summary of the published work on the properties of alumina-based nanoeutectic ceramics and their applications is presented in Table 1; it is evident that significant efforts have been invested to develop a family of nanocrystalline alumina-based eutectic ceramics, generally including Al_2_O_3_-YSZ, Al_2_O_3_-GdAlO_3_, Al_2_O_3_-GdAlO_3_-YSZ, Al_2_O_3_-Y_3_Al_5_O_12_, Al_2_O_3_-Y_3_Al_5_O_12_-YSZ, Al_2_O_3_-Er_3_Al_5_O_12_, Al_2_O_3_-Er_3_Al_5_O_12_-YSZ, etc. In particular, hardness, fracture toughness, wear, thermal stability, strength and creep, as well as thermal emission and optical transparency properties, have been studied. The main mechanical properties and emerging functional applications of DSE ceramics were reviewed by Hirano [107], LLorca et al. [96], Lakiza [98], Zhang et al. [108] and Orera [101]. Herein, we will only focus on the major recent developments of the mechanical aspects of alumina-based nanoeutectic ceramics.

### 5.1. Hardness, Fracture Toughness and Wear Resistance

**Figure 11:**
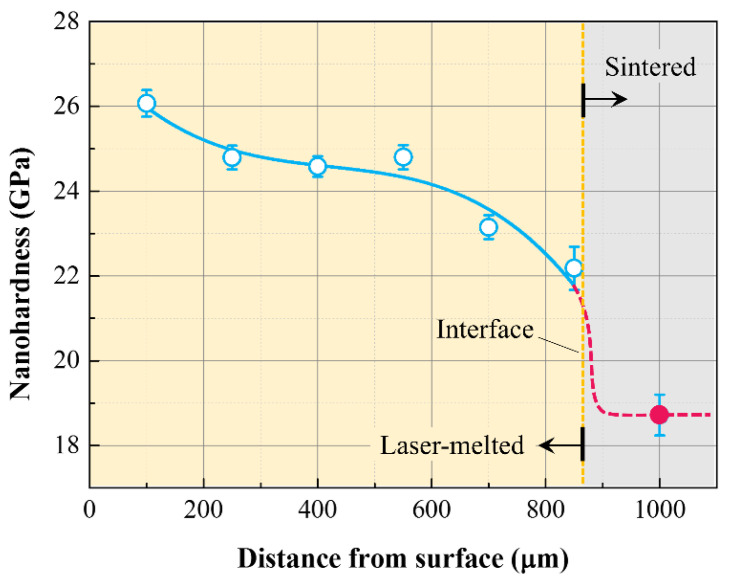
Nanohardness distribution of the nanostructured Al_2_O_3_-YSZ eutectic layer induced by the rapid laser remelting process as a function of the distance from the surface. Reproduced with permission from Reference [66], Copyright © 2019 Elsevier Ltd. and Techna Group S.r.l.

Hardness of DSE oxides is primarily a function of the hardness of the constituent eutectic phases, and the Al_2_O_3_-ZrO_2_(Y_2_O_3_) eutectic, labelled as Al_2_O_3_-YSZ, exhibits the highest values reported in the literature, reaching more than 23.5 GPa when the doped content of Y_2_O_3_ reached 1.5 mol% [109]. Fan et al. [51] reported that the hardness value of Al_2_O_3_-YSZ eutectic ceramics with spacing of about 250 nm, formed via LFZ, was in the range of 15–16 GPa. Lee et al. [23] found that the hardness value increased from 11 to 13.1 GPa as the lamellar thickness decreased from 380 to 110 nm, which indicated that decreasing eutectic spacing has an advantage for hardness enhancement. In addition, Fan et al. [78] reported that Al_2_O_3_-YAG-YSZ nanoeutectic ceramics (spacings of less than 500 nm) presented hardness, measured via Vickers indentation, of about 18.9 GPa, which is comparable to or even higher than those of most DSE oxides, which have hardnesses in the range of 12–18 GPa. In addition, no distinct difference was observed for transverse or longitudinal sections, with 18.9 GPa for the longitude section and 18.7 GPa for the transverse section, respectively, which indicates the isotropic hardness properties. The hardness of Al_2_O_3_-GdAlO_3_-YSZ nanoeutectic ceramics reported in the literature [25,26] is 16–18 GPa, which is comparable to that of the Al_2_O_3_-GdAlO_3_-YSZ system [27,82,83]. The relationship between microstructure and hardness in eutectic ceramics with colony microstructures is more complicated to assess, but nanostructuring of the fibers or lamellae within the colonies can indeed improve hardness. This effect was mainly ascribed to dislocation strengthening induced by the presence of interface domains. From Figure 11, it is evident that a substantial hardness enhancement is achieved in the Al_2_O_3_-YSZ nanoeutectic layers, where the outermost nanoeutectic surface layer exhibited a remarkably high nanohardness of 26.1 GPa, as contrasted with that (18.7 GPa) of the inner eutectic zone with a coarsened microstructure [66].

The fracture toughening and mechanisms of alumina-based nanoeutectic ceramics with the best mechanical properties of the Al_2_O_3_-YSZ system have received special attention in the literature [47,86,87,110]. However, the experimental findings reported in the literature are scarce and not yet convincing enough due to the limited availability of defect-free samples large enough to carry out the measurement. Consequently, fracture toughening of alumina-based nanoeutectic ceramics is mainly measured using the Vickers indentation method. The indentation fracture (IF) toughness of Al_2_O_3_-YSZ nanoeutectic ceramics produced with combustion synthesis reached up to 10.6 ± 0.49 MPa·m^1/2^ [87] as compared to the 5–8 MPa·m^1/2^ of other reported results, which seems to be the limit for alumina-based nanoeutectic ceramics. The improvement of the fracture toughness was speculated to be related to refined eutectic spacing and residual compressive stress. In addition, Al_2_O_3_-GdAlO_3_ presented IF toughness in the range of 3–5 MPa·m^1/2^ [25,26], while ternary Al_2_O_3_-GdAlO_3_-YSZ [27] eutectic ceramics showed enhanced values of 6.1 to 7.8 MPa·m^1/2^. Fan et al. [63,78] found that the IF toughness of Al_2_O_3_-Y_3_Al_5_O_12_ was 3.7 MPa·m^1/2^, which is apparently lower than that of the ternary Al_2_O_3_-Y_3_Al_5_O_12_-YSZ system (5.6 MPa·m^1/2^) [111]. The Al_2_O_3_-Er_3_Al_5_O_12_ nanoeutectic ceramics presented a low IF toughness of 1.9 MPa·m^1/2^ [30], which could be improved up to 3.5 MPa·m^1/2^ with the introduction of 20.6 mol% YSZ [50]. The improvement of fracture toughness was mainly ascribed to the crack bridging and deflection induced at phase interfaces as well as the phase-transformation toughening produced by the YSZ phases.

The friction and wear resistance of alumina-based nanoeutectic ceramics have been investigated mainly in the Al_2_O_3_-YSZ system. Generally, the friction coefficients were high in all cases of laser-melted surfaces [58], laser-engineered net shaping bulks [75] and thermal-explosion-sprayed coatings [58], as expected from unlubricated wear. The wear rate of the laser-melted surfaces presented a range from 2 × 10^−6^ to 1 × 10^−5^ mm^3^·N^−1^m^−1^, and the coefficient of friction was found to be 0.45–0.60, which is significantly superior to that of sintered polycrystalline ceramics. Clearly, the laser surface melting nanoeutectic layer provides a promising method in significantly enhancing hardness and wear resistance for applications as sliding ceramic components.

### 5.2. Flexural and Tensile Strength

**Figure 12:**
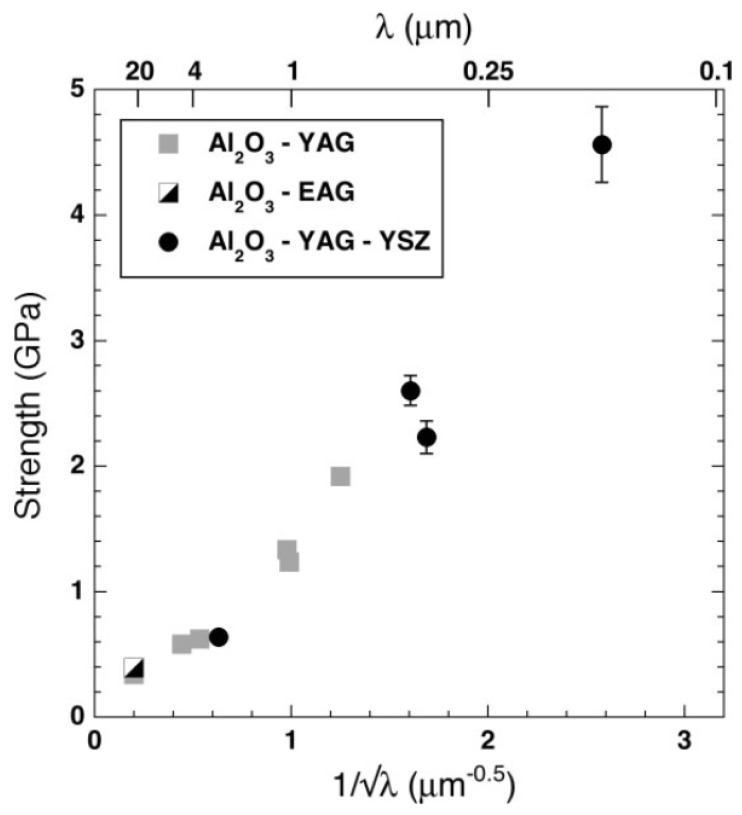
Influence of average domain size, λ, on the flexure strength of bulk specimens of directionally solidified eutectic oxides. Reproduced with permission from Reference [13], Copyright © 2007 WILEY-VCH Verlag GmbH & Co. KGaA, Weinheim.

The characteristic microstructures of three-dimensionally interwoven nanoscale eutectic phases with atomically clean and strongly cohesive interfaces are of decisive significance in determining the mechanical behavior of alumina-based nanoeutectic ceramics. For example, Fan et al. [51] found that the flexural strength of Al_2_O_3_-ZrO_2_ nanoeutectic ceramic (300 nm in spacing) rods produced with the LFZ method could reach up to 1.4 GPa, which is close to the best flexure strength, 1.6 GPa, measured in rods with lamellar microstructures [38]. In addition, Al_2_O_3_-YAG-YSZ nanoeutectic ceramic rods showed exceptional mechanical resistance in air at up to temperatures above 2000 K [1,13] and a three-point bend strength of 4.6 GPa, which is approximately twice as high as the highest reported in DSE oxides of the Al_2_O_3_-YAG-YSZ family, as shown in Figure 12.

**Figure 13:**
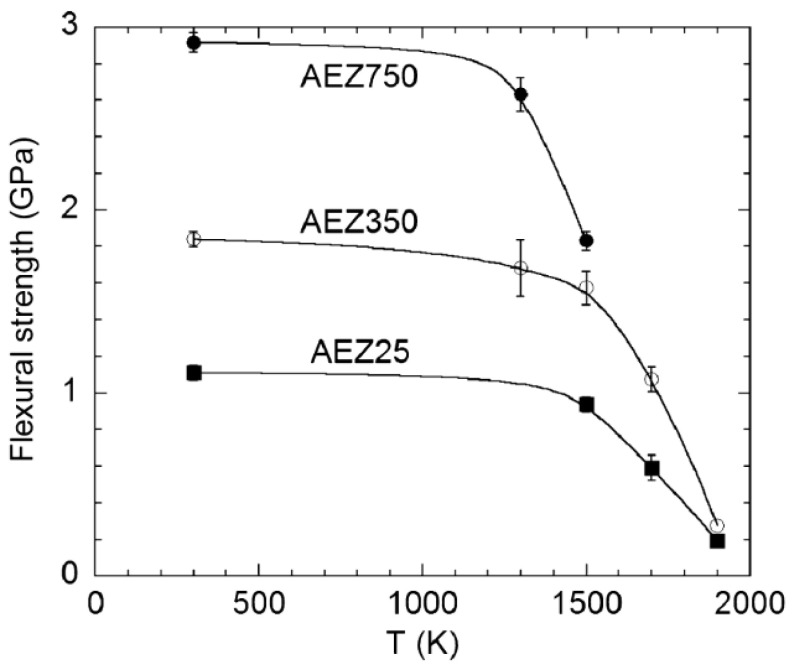
Flexural strengths of AEZ25 (25 mm/h), AEZ350 (350 mm/h) and AEZ750 (750 mm/h) as a function of temperature. Reproduced with permission from Reference [47], Copyright © 2018 Elsevier Ltd.

Pena’s results also demonstrated that the Al_2_O_3_-YAG-YSZ ternary eutectic showed excellent strength retention (2.2 GPa) up to 1473K, but the flexure was reduced to one-half at 1700 K [16], which is still comparable to the best binary eutectics of the Al_2_O_3_-YAG system. In addition, Mesa’s results [47] indicated that the flexural strength of Al_2_O_3_-EAG-YSZ increased from 1.1 GPa up to 2.9 GPa as the EAG domains decreased from 1.6–2.5 μm to 0.15–0.22 μm; especially, high strength retention was observed at up to 1500 K. Above this temperature, the flexural strength showed an abrupt reduction, as shown in Figure 13. The flexural strength of an Al_2_O_3_-GdAlO_3_ eutectic rod could reach a maximum value of 1.14 GPa and could be further improved to 1.78 GPa through refining of the eutectic microstructure. The origin of this enhanced strength can be traced to nanostructured eutectic domains, if the microstructure is homogeneous, and to improvement in resistance to crack propagation perpendicular to elongated oxide monocrystalline domains. However, those authors observed that the strength increased from 0.96 to 1.36 GPa as the eutectic spacing decreased from 2500 to 300 nm, and then dramatically decreased to 0.79 GPa, with further decreasing of the spacing below 250 nm [51]. This decline of the flexure-strength value may be mainly ascribed to cracking defects induced by residual stress during rapid solidification due to the brittle nature of ceramics and their susceptibility to cracking defects.

**Figure 14:**
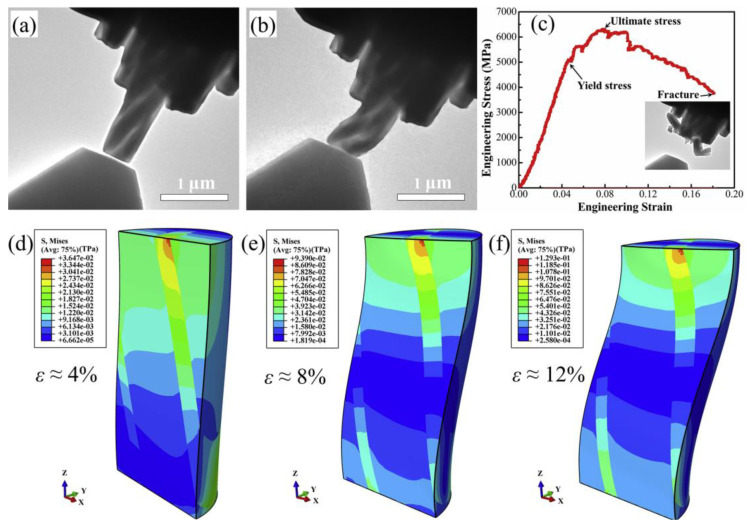
Micropillar compression test of rod-like Al_2_O_3_-GdAlO_3_ nanoeutectic with rods oriented toward the compression axis: (**a**) bright-field TEM image before deformation, (**b**) bright-field TEM image after deformation, (**c**) the corresponding engineering stress-strain curves and (**d**–**f**) FEM simulation of the stress-field distribution at different engineering strains. Reproduced with permission from Reference [113], Copyright © 2020 Elsevier Ltd.

The room-temperature tensile strength of Al_2_O_3_-ZrO_2_ eutectic fibers produced with the micro-pulling-down method increased from 470 to 900 MPa as the lamellar thickness decreased from 380 to 110 nm [23]. Sayir et al. [34] proved that the Al_2_O_3_-YSZ nanoeutectic ceramic does not exhibit steep strength losses at elevated temperatures of up to 1500 °C, which can be attributed to the nanostructuring effect induced by the increasing pulling rate. Zhai et al. [7] found that the value of 1500 °C tensile strength of Al_2_O_3_-YSZ eutectic ceramics increased dramatically from 55.2 MPa to 94.3 MPa, with decreasing interphase spacing from 5.2 to 2.2 μm. The room-temperature tensile strength was 172.2 MPa, with an interphase spacing of 4.6 μm. Clearly, these results provide solid experimental evidence that a finer eutectic microstructure is beneficial for enhancing tensile strength. For example, nanofibrillar Al_2_O_3_-YAG-ZrO_2_ eutectic rods with YAG whiskers of about 100 nm in width exhibited superplastic behavior at 1600 K and above [45]. Compressive deformation tests of Al_2_O_3_-YAG-ZrO_2_ performed at 1400 °C at a constant initial strain rate in air indicated that the creep resistance of the eutectics decreased with the increasing growth rate due to the refinement of the interlamellar spacing [112]. More recently, uniaxial micropillar compression tests were conducted on nanostructured Al_2_O_3_-GdAlO_3_ eutectic ceramics; the presence of a heterogeneous interface drove elastic-strain inhomogeneities, which caused buckling and catastrophic failure of these submicron pillars when compressed in certain eutectic lamellar orientations, as demonstrated in Figure 14 [113].

### 5.3. Summary of Mechanical Properties

In the case of nanocrystalline alumina-zirconia-based eutectic ceramics formed with high-energy beams, when a rapid solidification process generally accompanies the process, this often leads to cracking due to large thermal gradients imposed by extremely localized heating from the high-energy density beams, which has imposed great limitations on these ceramics’ scientific research and practical applications. Therefore, the most currently published findings have mainly focused on investigating mechanical properties, such as Vickers hardness and indentation-fracture toughness, via laboratory tests in small-scale samples. Combined with surface mechanical properties and micromechanical behaviors, these results have provided general evidence that decreasing the characteristic microstructural dimension (or refinement), including either the colony size or eutectic interspacing, can indeed improve the mechanical performance of alumina-zirconia-based eutectic ceramics as a result of reduction in typical defect size.

## 6. Conclusions and Perspectives

The comprehensive review of the developments in nanocrystalline alumina-zirconia-based eutectic ceramics presented in this paper exhibits that they are particularly intriguing multiphase ceramic composites with three-dimensionally entangled ultrafine structures and clean and strongly cohesive interfaces, which makes them potential candidates for high-temperature mechanical and structural applications in hot-section components of gas generators or aerospace jet engines. As of yet, some aspects of remarkable progress have been achieved, particularly in recent years, and a number of key issues remain open regarding processing, microstructure, mechanical properties and applications of alumina-zirconia-based nanoeutectic ceramics. The last section of this review concludes by briefly pointing out achievements and shortcomings, and necessary breakthroughs, as well as emerging areas of research, are noted.

The following conclusions have been drawn:

(1) Unveiling the undercooling-growth rate relationship, in particular, would lead, via the coupled zone concept, to a better understanding of how to control the eutectic microstructure. Based on this, possible contributions involved in the evolution of growth undercooling are elucidated in this review, and the *V*-*λ*-Δ*T* relationship is presented with regard to rapid solidified regular and irregular eutectic ceramics. Excellent agreement was achieved between the experimental results and the theoretical models.

(2) Due to the intrinsically high melting points, low thermal and electrical conductivities and high melting entropy of alumina-zirconia-based composites, achieving bulk crack-free and highly dense eutectic ceramics is still a big challenge worldwide. A potential major step toward remedying the situation is to develop a high-energy beam-based melting process. Laser-heated float zoning, high-energy-beam surface melting and directed energy deposition, in which larger thermal gradients and consequently faster growth rates can be attained, are the current prevailing approaches for producing alumina-based nanoeutectic ceramics. Among these methods, the laser-heated float-zoning process is suitable for growth of eutectic ceramic fibers, and directed energy deposition has potential to prepare, in one step, ceramic parts with complex shapes. High-energy-beam surface melting is mainly applied to produce in situ surface nanoeutectic ceramics.

(3) For rapidly solidified alumina-zirconia-based eutectic ceramics, due to microsegregation of the chemical compositions and creation of nonequilibrium phases, the eutectic microstructure is characterized mainly by cellular eutectic grains. In fact, colony structure is regarded as the critical defect. In this regard, another challenge is the difficulty in producing the planar and stable solidification fronts necessary for fine and homogeneous eutectic microstructures. Imposing a large growth rate or an ultrasonic vibration, to some degree, can prevent or alleviate the occurrence of eutectic colonies.

(4) The growth of alumina-based nanoeutectic ceramics is normally accompanied by the appearance of large thermal residual stresses and a coarse inter-colony region, which significantly deteriorates the mechanical and functional properties of the ceramics due to a tendency to crack and trap bubbles within the coarse inter-colony region. To avoid cracking induced by thermal shock, one of the obvious strategies is to perform the preheating process of the substrate to be treated to reduce thermal stress. In addition, it will be a persistent goal to explore the microstructural evolution and formation mechanisms of nanoeutectic ceramics.

(5) Nanostructured, alumina-based eutectic ceramics indeed exhibit enhanced high-temperature flexural and tensile strength, as well as hardness and wear resistance, compared to DSE oxides. However, due to the limited availability of defect-free and large-scale bulk alumina-based nanoeutectic ceramics, it is necessary for further research to solve some key technical issues related to microstructural tailoring and feasible production technology for a step forward toward practical applications.

(6) Generally speaking, alumina-zirconia-based eutectic ceramics exhibit great application potential in the field of surface engineering, such as improving wear resistance, repairing localized manufacturing defects, sealing surface pores, texturing surface layers, producing functional coatings on ceramic substrates, etc. In addition, alumina-zirconia-based eutectic ceramics can be applied as nanoeutectic fibers or particles to form reinforced composites and reach their full potential in terms of superb oxidation and creep resistance as well as mechanical resistance at elevated temperatures.

## Figures and Tables

**Figure 8:**
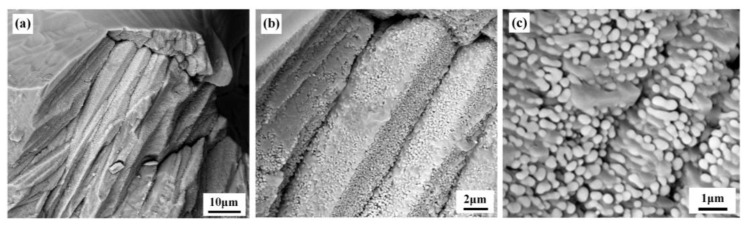
Back-scattered electron images showing three-dimensional configurations of faceted nanocrystalline Al_2_O_3_-ZrO_2_ eutectic grains with competitive growth, produced through oxy-acetylene flame remelting. (**b**) Single-faceted grains oriented parallel to each other in (**a**). (**c**) Typical ZrO_2_ rods distributed in the cores of grains. Reproduced with permission from Reference [65], Copyright © 2018 The American Ceramic Society.

**Table 1:** Summary of the published work on the properties of alumina-based nanoeutectic ceramics and their applications.

Material	Hardness	Fracture Toughness	Wear Resistance	Thermal Stability	Flexural Strength	Tensile Strength	Creep Properties	Thermal Emission	Optical Transparency	Applications	Refs.
Al_2_O_3_-YSZ	○○○○○○○○○○○	○○○○○○○○	○○○	○	○○○	○○	○			Structural	[8,22,23,34,36,38,51,58,60,70,74,75,86,87,109,110]
Al_2_O_3_-GdAlO_3_	○○	○○		○	○					Structural	[25,26]
Al_2_O_3_-GdAlO_3_-YSZ	○○	○○		○						Structural	[27,82,83]
Al_2_O_3_-Y_3_Al_5_O_12_	○○○	○○						○		Electromagnetic	[62,63]
Al_2_O_3_-Y_3_Al_5_O_12_-YSZ	○	○			○				○	Optical	[13,59,78]
Al_2_O_3_-Er_3_Al_5_O_12_	○	○			○					Structural	[30]
Al_2_O_3_-Er_3_Al_5_O_12_-YSZ	○○	○○		○○	○					Structural	[47,48,50]

The number of “○” symbols indicates the frequency of investigation.

## Data Availability

Not applicable.
